# Synergy between stem cell therapy and brain-derived neurotrophic factor (BDNF) in Parkinson’s disease: a mini-review of combined neuroregenerative strategies

**DOI:** 10.1186/s41016-025-00415-5

**Published:** 2025-11-06

**Authors:** Jonny Jonny, Astrid Devina Larasati, Queenesa Amabel Sunjaya, Ahmad Faried

**Affiliations:** 1 Faculty of Military Medicine, Indonesia Defense University, Bogor, Indonesia; 2 Faculty of Medicine, Dentistry, and Health Sciences, Prima Indonesia University, Medan, Indonesia; 3 Department of Internal Medicine, Nephrology Division, Gatot Soebroto Central Army Hospital, Jakarta, Indonesia; 4 Faculty of Medicine, Padjadjaran University, Bandung, Indonesia

**Keywords:** Parkinson, Stem cell, Brain-derived neurotrophic factor, Dopaminergic neurons, Neuroregeneration

## Abstract

Parkinson’s disease remains a progressive and debilitating neurodegenerative disorder with limited therapeutic options that can modify disease progression. While conventional treatments like levodopa alleviate motor symptoms, they often fall short in addressing long-term neurodegeneration and may lead to significant side effects. Recent advances in regenerative medicine have highlighted the potential of combining stem cell therapy with Brain-Derived Neurotrophic Factor (BDNF) enhancement as a synergistic approach to restore dopaminergic function and promote neuronal survival. Stem cells not only offer the capacity to replace lost neurons but can also serve as delivery vectors for sustained BDNF expression, amplifying neuroprotective effects through Tropomyosin receptor kinase B-mediated signaling pathways. Preclinical studies in animal models demonstrate that this combined strategy enhances motor recovery, reduces neuroinflammation, and promotes neural circuit integration. As the field progresses, this dual therapy holds great promise for transforming the future management of Parkinson’s disease by offering both symptomatic relief and disease modification.

Parkinson’s disease (PD) is a slowly progressive neurodegenerative disorder, primarily affecting the elderly. This condition results from damage to dopamine-producing neurons in the substantia nigra of the brain and the accumulation of abnormal proteins that accelerate neuronal degeneration [1]. Initial symptoms often manifest as mild tremors, progressing to bradykinesia, rigidity, and eventually postural instability, which significantly interferes with daily activities [2, 3]. In addition to motor symptoms, PD is characterized by non-motor disorders, including impaired olfactory function, sleep disturbances—particularly in the Rapid Eye Movement (REM) phase—mood changes, drooling, constipation, and autonomic dysfunction [4]. Some non-motor symptoms may precede motor deficits, highlighting their importance in early detection [5].

The Global Burden of Disease Study indicates a significant increase in PD cases, disability, and mortality worldwide [6]. Global prevalence is approximately 1.51 per 1,000 population, with higher incidence in men than women [7]. In 2021, 1.34 million new cases were reported globally, and the total number of patients increased by 274% since 1990, from 3.15 million to 11.77 million cases [8]. In Indonesia, prevalence in 2019 was 89.91 per 100,000 people, representing a 143% increase since 1990 [9]. Although direct mortality data are limited, PD patients have a 1.5-fold higher risk of death than the general population [10].

Current treatments focus on symptom management, including pharmacological interventions and surgical procedures. The primary pharmacological therapy involves levodopa, which is converted into dopamine in the central nervous system to alleviate motor deficits [11]. Levodopa is commonly combined with carbidopa, an aromatic amino acid decarboxylase inhibitor, to reduce side effects and allow lower effective doses [12]. Sudden discontinuation of levodopa can cause serious complications, including mobility impairment and respiratory difficulties [13]. Long-term use may lead to dyskinesia and neuropsychiatric disorders, contributing to treatment discontinuation in approximately 34% of patients after four years [2, 14].

Given the limitations of conventional therapies, alternative approaches are increasingly explored [15]. Emerging strategies include stem cell transplantation and enhancing Brain-Derived Neurotrophic Factor (BDNF) levels to support dopaminergic neuron survival [16]. Stem cell therapy aims to restore motor function by implanting progenitor cells capable of differentiating into dopamine-producing neurons, potentially replacing damaged cells [17]. Simultaneously, elevated BDNF is believed to promote neuron survival and regeneration, exerting neuroprotective effects that may improve clinical symptoms [18]. Therefore, this review focuses on evaluating the combined use of stem cell therapy and BDNF modulation as an integrated approach for Parkinson’s disease treatment.

## Overview of Parkinson’s disease

Parkinson’s disease (PD) is a progressive neurological disorder characterized by motor symptoms including resting tremor, rigidity, bradykinesia, and postural instability, resulting from dopaminergic neuron degeneration in the nigrostriatal pathway [19, 20]. PD is part of the broader spectrum of parkinsonism, classified into primary and secondary types. Primary parkinsonism includes idiopathic PD—sporadic or familial—and atypical variants such as progressive supranuclear palsy, multiple system atrophy, corticobasal degeneration, and Lewy body dementia. Secondary parkinsonism arises from identifiable external factors, including drugs, toxins, vascular events, or traumatic brain injury [21]. Epidemiological data show that idiopathic PD accounts for 85–90% of cases, with atypical and secondary forms being less common [22, 23]. Technological advances, including smart diagnostic pens, machine learning applications for gait analysis, and Magnetic Resonance Imaging (MRI), offer promising tools for early detection and differentiation of PD from other neurodegenerative diseases [24–26].

PD is currently one of the fastest-growing neurological disorders globally in terms of prevalence, disability, and mortality [27]. Global prevalence is approximately 1.51 per 1,000 people, with higher rates in men [7]. In 2021, 1.34 million new cases were reported, and total cases increased 274% from 3.15 million in 1990 to 11.77 million in 2021 [8]. In Low-and Middle-Income Countries (LMICs), prevalence can reach 516 per 100,000, influenced by GDP per capita and life expectancy [28]. In Indonesia, prevalence in 2019 was 89.91 per 100,000, a 143% increase since 1990 [9]. Globally, PD accounted for 5.8 million Disability-Adjusted Life Years (DALYs) and 329,000 deaths in 2019, with DALYs increasing by 81% and mortality doubling since 2000 [29]. While no definitive disease-specific mortality exists, PD patients have a 1.5-fold higher risk of death than the general population [10]. Additionally, prevalence patterns in LMICs, including Indonesia, often surpass global averages [30, 31].

Clinically, PD presents with both motor and non-motor symptoms. Motor features include tremor, bradykinesia, rigidity, and postural instability, while non-motor symptoms encompass cognitive decline, mood disorders, sleep disturbances, autonomic dysfunction, and sensory impairments such as reduced olfaction [32, 33]. These diverse manifestations underline the complexity of PD and the challenges in comprehensive management.

Pathophysiologically, major changes occur in the brainstem, with substantial depigmentation of the Substantia Nigra pars compacta (SNpc) and locus coeruleus, correlating with selective loss of dopaminergic neurons containing neuromelanin [34]. Postmortem studies show approximately 30% dopaminergic neuron loss at motor symptom onset, progressing to over 60% in advanced stages, causing marked striatal dopamine depletion [35]. This dopaminergic imbalance disrupts the equilibrium of basal ganglia circuits, contributing to motor dysfunction [36, 37]. Additionally, neuronal loss in other subcortical regions and involvement of non-dopaminergic neurotransmitter systems contribute to non-motor symptoms [38].

### Current treatments for Parkinson’s disease

Current treatments for Parkinson’s disease remain largely symptomatic [19], often requiring multiple drugs with significant side effects. These therapies address symptoms without targeting underlying neuronal degeneration. Current therapeutic approaches for Parkinson’s disease aim to alleviate motor symptoms, delay disease progression, and improve patients’ quality of life through pharmacological, device-assisted, and surgical strategies. These therapies primarily target dopamine deficiency and abnormal protein accumulation, with approaches ranging from conventional levodopa-based regimens to advanced neuromodulation and continuous drug delivery systems. A comprehensive summary of these treatment modalities, including their mechanisms, advantages, and limitations, is presented in Table 1.

**Table 1:** Current treatment strategies for Parkinson’s disease

Strategy/Therapy	Mechanism of Action	Advantages	Limitations/Side Effects
**Targeting α-synuclein** (reducing buildup, promoting clearance, inhibiting LRRK2, enhancing cerebrosidase activity)	Modulate α-synuclein accumulation and related pathways	Potential disease-modifying effect	Mostly preclinical/experimental, limited clinical translation [11]
**Levodopa**	Dopamine precursor; restores dopamine levels in the brain	Gold standard; improves motor symptoms and quality of life [39, 40]	Long-term use → dyskinesia [41]; reduced responsiveness over time; requires higher/frequent dosing [42]
**Levodopa + Decarboxylase inhibitors** (Carbidopa, Benserazide)	Prevent peripheral metabolism of levodopa, increase CNS bioavailability	Enhance levodopa efficacy; reduce complications [43]	Side effects still possible; not curative
**Dopamine agonists** (e.g., Ropinirole, Pramipexole, Rotigotine patches, Apomorphine)	Directly stimulate dopamine receptors	Effective as monotherapy or with levodopa [12]; continuous delivery with patches [40, 40]; apomorphine useful as rescue therapy [44]	Nausea, hypotension, somnolence, impulse control disorders; apomorphine requires injection/infusion
**Deep Brain Stimulation (DBS)**	Modulation of abnormal basal ganglia activity via implanted electrodes	Reduces “off-time,” improves motor function and quality of life [45, 46]	Surgical risks (seizures, neuropsychiatric effects), invasive, expensive
**Levodopa-Carbidopa enteral suspension**	Continuous high-dose levodopa via gastrostomy tube	Stabilizes plasma levels; reduces motor complications [12]	High cost; side effects; limited patient adherence [14]
**Continuous apomorphine infusion**	Non-surgical continuous dopaminergic stimulation	Provides stable drug delivery; reduces levodopa need [12]	Efficacy decreases over time; frequent side effects → high dropout rates [47]

As shown in Table 1, Parkinson’s disease management encompasses a spectrum of pharmacological and advanced therapeutic strategies. While levodopa remains the cornerstone of treatment, long-term complications such as dyskinesia and motor fluctuations highlight the importance of adjunctive medications and device-assisted therapies. Advanced interventions, including deep brain stimulation and continuous infusion systems, provide additional options for patients unresponsive to oral pharmacotherapy. Nevertheless, these approaches are often limited by invasiveness, high cost, and adverse effects, underscoring the need for novel strategies with greater efficacy and safety.

### Cell replacement therapy

Stem cell therapy has emerged as a promising alternative, offering cell replacement, immune modulation, and neurotrophic support [48]. Stem cells, such as Human Pluripotent Stem Cells (hPSCs), can be differentiated into midbrain Dopaminergic Progenitors (mDAPs) and transplanted to restore dopamine levels and improve motor function [49–51]. These cells integrate into host circuits, release dopamine physiologically, and promote neurorestoration, though challenges like tumorigenesis, immune response, and graft-induced dyskinesia remain [52].

Stem cells are a type of cell with the unique ability to self-renew and differentiate into multiple cell lineages. They have shown initial promise in treating neurodegenerative diseases such as Parkinson’s disease [53]. Stem cell classified based on two group, the first one based on differentiation, such as totipotent, pluripotent, multipotent, oligopotent and unipotent. The second one based on origin, such as embryo (embryonic stem cells and embroyonic germ stem cells), fetus, infant, and adult [54]. Recent advancements in stem cell research have introduced promising therapeutic alternatives for Parkinson’s disease patients unresponsive to conventional treatments, by generating patient-specific Pluripotent Stem Cells (PSCs) capable of differentiating into various functional cell types both in vitro and in vivo [52]. Stem cell therapy has the ability to differentiate into neural cells and can be induced to acquire specific functions, making it a potential therapeutic option for Parkinson’s disease. Candidates for Parkinson’s therapy include embryonic stem cells, adult mesenchymal stem cells, and induced pluripotent stem cells [55]. Midbrain dopaminergic neurons for striatal transplantation can be derived from multiple sources, including human fetal tissue, porcine-derived fetal substantia nigra neurons, carotid body-derived cells, and progenitor retinal cells. Pluripotent Stem Cells (PSCs), including Embryonic Stem Cells (ESCs) and induced Pluripotent Stem Cells (iPSCs), are capable of differentiating into a broad range of cell types, including neurons exhibiting dopaminergic traits [56].

Cell replacement for Parkinson’s disease replaces the dopamine neurons lost to degeneration and can restore dopaminergic function in the brain [57]. Mechanisms of stem cell therapy in Parkinson’s disease can be classified into two categories. The first is the direct repair pathway, which includes augmenting endogenous neurogenesis, Dopaminergic (DA) neuron differentiation, DA release, striatal reinnervation, and neural circuit integration. The second is the indirect repair mechanism, which involves the action of neurotrophic factors—such as Brain-Derived Neurotrophic Factor (BDNF), Nerve Growth Factor (NGF), Cerebral Dopamine Neurotrophic Factor (CDNF), and Glial cell line-Derived Neurotrophic Factor (GDNF)—to facilitate DA neuronal differentiation and support neuronal survival and maintenance [58]. Differentiation is directed or modified through genetic engineering of Neural Stem Cells (NSC), which are then transplanted into the central nervous system related to the target system, or placed in stationary regions of the brain associated with the induction and activation of NSC [59].

Recent clinical studies further highlight the translational potential of stem cell therapy in PD. Personalized iPSC-derived dopaminergic progenitor cells have been generated for patient-specific regenerative therapy [60]. In a Phase I/II trial, iPSC-derived dopaminergic progenitors successfully engrafted, released dopamine, and showed no tumorigenesis [61]. Similarly, hESC-derived dopaminergic neurons were safely transplanted in a Phase I trial, integrated into host neural circuits, and contributed to functional recovery [62]. These findings underscore the feasibility and promise of stem cell-based neuroregenerative strategies in Parkinson’s disease.

Stem cell application is associated with both significant benefits and notable limitations. iPSCs offer a notable advantage in enabling autologous transplantation, as the derived cells are genetically matched to the donor, thereby substantially reducing the risk of immune rejection [56]. An meta-analysis provides preliminary evidence for the beneficial effects of homogenous cell-therapy for Parkinson Disease, potentially to the levodopa responders [15]. While stem cell therapy shows promise for PD, challenges such as patient selection, graft composition, and immune response need addressing [58]. Stem cell treatment remains highly promising and may be further enhanced by combining it with gene therapy. Nevertheless, it has several limitations, including the absence of a reliable differentiation system, limited efficiency in differentiation, potential for tumor formation, uncertain safety, and an incomplete understanding of its specific therapeutic mechanisms [63]. Optimization of stem cell therapy for PD can be done by: 1) choosing the right donor stem cells, 2) inducing and selecting DA neurons appropriately, 3) conducting valid preclinical research, 4) optimizing the host brain environment [64].

## The role and therapeutic potential of BDNF in Parkinson’s disease

Brain-Derived Neurotrophic Factor (BDNF) supports dopaminergic neuron survival and regeneration, enhancing motor control [18, 65]. BDNF has shown therapeutic potential in Parkinson’s Disease, but is limited by its short half-life and inability to penetrate the Blood–Brain Barrier (BBB) [66]. It can be delivered directly or via genetically engineered stem cells (MSCs or iPSCs) to continuously secrete BDNF after transplantation [19, 67]. Combining stem cell therapy with BDNF provides a potential integrated strategy to address both symptomatic relief and underlying disease mechanisms in Parkinson’s disease.

Brain-Derived Neurotrophic Factor (BDNF), a neurotrophin family growth factor, supports neuronal survival, differentiation, and maintenance in the central nervous system [18, 68]. It regulates neuronal differentiation and synaptic plasticity—processes essential for learning and memory—throughout life [69]. BDNF exists in three isoforms: pro-BDNF, BDNF prodomain, and mature BDNF, with mature BDNF being the active form that binds to Tropomyosin receptor kinase B (TrkB) to promote dendritic growth, synaptic plasticity, and neuronal survival [70].

In Parkinson’s disease, BDNF maintains dopaminergic neuron function in the substantia nigra pars compacta [71]. A loss of GABAergic (Gamma-Aminobutyric Acid) neurons in the substantia nigra contributes to Parkinson’s pathology, and BDNF protects them from excitotoxicity [72]. BDNF also regulates dopamine signaling by modulating dopamine receptors and tyrosine hydroxylase, a key enzyme in dopamine synthesis [73]. BDNF activates intracellular signaling through TrkB, triggering Phosphoinositide 3-Kinase/Protein Kinase B (PI3K/Akt), Mitogen-Activated Protein Kinases/Extracellular Signal-Regulated Kinases (MAPK/ERK), and Phospholipase C Gamma (PLC-γ) pathways [18, 74]. These pathways protect neurons by enhancing survival, synaptic plasticity, and reducing apoptosis and oxidative stress—mechanisms relevant to PD [71]. BDNF is expressed in SNpc dopaminergic neurons (~ 70% co-express BDNF/TrkB) and other motor-related regions like basal ganglia, cerebellum, and brainstem, with levels reduced in PD patients correlating with motor deficits [75, 76].

Preclinical studies show that exogenous BDNF or upregulation of endogenous BDNF reduces dopaminergic neuron loss and improves motor function in PD models [18, 77]. BDNF also modulates inflammation and mitochondrial function, further supporting its therapeutic potential [74]. BDNF increases dopamine release and uptake, while TrkB deficiency leads to neuron loss and α-synuclein buildup, suggesting that enhancing BDNF signaling may offer new treatment options [74].

However, BDNF therapy still faces challenges in PD treatment.

Despite success in animal models, clinical trials struggle with administration and delivery stability [78]. Direct administration or gene therapy has shown limited clinical outcomes [18]. BDNF’s large size prevents it from crossing the blood–brain barrier, requiring invasive delivery that risks seizures or Schwann cell migration [79, 80]. Targeted SNpc delivery remains unreliable and may cause systemic or local side effects, such as cell migration, inflammation, and seizures. Thus, new delivery methods like nanoparticles, ex vivo gene therapy, and systemic routes are being developed [81]. Other hurdles include the need for prolonged treatment (over 18 months), local expression control (e.g., gene modulation or exercise), early-stage intervention, and combination therapy [82]. Combining BDNF with stem cell therapy is now being studied to promote neuroregeneration and functional recovery in PD.

## Combining cell replacement therapy and BDNF

Stem cell therapy for Parkinson’s disease aims to replace degenerated dopaminergic neurons via differentiation and functional integration of transplanted stem cells into the host tissue. Transplanted pluripotent-derived stem cells can be guided to differentiate into dopaminergic progenitor cells expressing key transcription factors; Nuclear receptor-related factor1 (Nurr1), Paired-like homeodomain3 (Pitx3), and LIM homeobox transcription factor 1 alpha (Lmx1a), which are essential for acquiring the midbrain dopamine neuron identity [77]. Overexpression of Nurr1 and Pitx3 in pluripotent stem cells has been shown to efficiently program them into functional dopaminergic-like neurons capable of synthesizing and secreting dopamine. In vitro, the simultaneous expression of Nurr1 and Pitx3—achieved through inducible systems or lentiviral vectors—can consistently produce Tyrosine Hydroxylase (TH)-positive neurons. These neurons are responsive to secretagogue stimulation, indicating that they are functionally active and capable of dopamine synthesis [83]. Lmx1a, often regulated via a Wingless-related integration site 1- LIM homeobox transcription factor 1 alpha (Wnt1-Lmx1a) autoregulatory loop, cooperates synergistically with Nurr1 and Pitx3 to promote midbrain dopaminergic neuron differentiation in embryonic stem cell models. This cross-regulatory network integrates extrinsic Wnt1 and Sonic Hedgehog (SHH) signals to drive DA lineage specification [84]. Differentiated dopaminergic progenitors upregulate tyrosine hydroxylase and other dopamine-synthetic enzymes under the control of Nurr1/Pitx3 transcriptional regulation, enabling dopamine production and release. Co-expression studies confirm increased TH expression and dopamine secretion in reprogrammed cells [85]. These dopaminergic-like cells also activate intracellular signaling cascades—including PI3K/Akt and MAPK/ERK pathways—through autocrine/paracrine secretion of neurotrophic factors such as BDNF and GDNF, thereby enhancing cell survival, synaptic plasticity, and anti-apoptotic defenses. Transplanted stem cells modified to express BDNF markedly increase p-Akt (PI3K/Akt) and downstream survival signaling, while GDNF from stem cells similarly promotes Akt activation and ERK signaling, reinforcing neuroprotection [86]. Simultaneously, stem cell–derived neurotrophic support downregulates neuroinflammatory signaling—particularly via suppression of Nuclear Factor Kappa B (NF-κB) pathway activity—which shifts microglial polarization toward the regenerative anti-inflammatory (M2 phenotype) and creates a less hostile environment for dopaminergic regeneration. MSC-derived GDNF notably inhibits NF-κB signaling in microglia, reducing pro-inflammatory cytokine release and inflammation-mediated damage [87].

BDNF and GDNF are produced in brain regions different from their target sites—BDNF is synthesized in the cortex and acts in the striatum, while GDNF is produced in the striatum and acts in the substantia nigra. The loss of either factor equally contributes to neuronal degeneration in Parkinson’s disease. Despite challenges such as limited blood–brain barrier permeability, enhancing BDNF or GDNF remains a promising therapeutic strategy [88]. Recent clinical studies have demonstrated that BDNF levels are significantly reduced in the substantia nigra and striatum of Parkinson’s disease patients, correlating with both motor impairment and non-motor symptoms [68, 89]. A large meta-analysis also confirmed that BDNF levels are lower in PD patients compared with healthy controls, and are further decreased in PD patients with comorbid depression [68]. Moreover, interventional clinical trials have shown that exercise elevates circulating BDNF levels and improves both motor and non-motor outcomes in PD patients [90]. These findings strongly support the prioritization of BDNF as a therapeutic target in current Parkinson’s disease management strategies.

BDNF is increasingly preferred over GDNF in various Parkinson’s disease therapy models due to its broader neuroprotective profile and practical advantages in delivery [74]. BDNF acts via TrkB receptors, which are widely expressed across various brain regions, offering a more extensive range of therapeutic impact compared to GDNF, which acts through GDNF Family Receptor alpha 1/Rearranged during Transfection (GFRα1/RET) receptors with more restricted expression [72]. In terms of delivery, BDNF demonstrates better compatibility with gene therapy vectors and stem cell modification platforms, while GDNF delivery remains technically challenging due to poor BBB permeability and localized side effects [81]. BDNF-modified stem cells have shown enhanced neuroprotective effects and synaptic repair in preclinical PD models, demonstrating stable expression and reduced inflammation [91]. Moreover, BDNF supports synaptic plasticity and neurogenesis, functions which extend beyond dopaminergic survival and are beneficial in addressing the widespread neurodegeneration seen in advanced PD [75]. Although GDNF has shown promising dopaminergic effects in specific settings, several clinical trials have reported inconsistent outcomes, partly due to difficulties in delivery and targeting [82]. Consequently, while GDNF remains valuable in dopaminergic regeneration, BDNF offers a more feasible and versatile therapeutic option in the current landscape of Parkinson's treatment development [74].

**Fig. 1:**
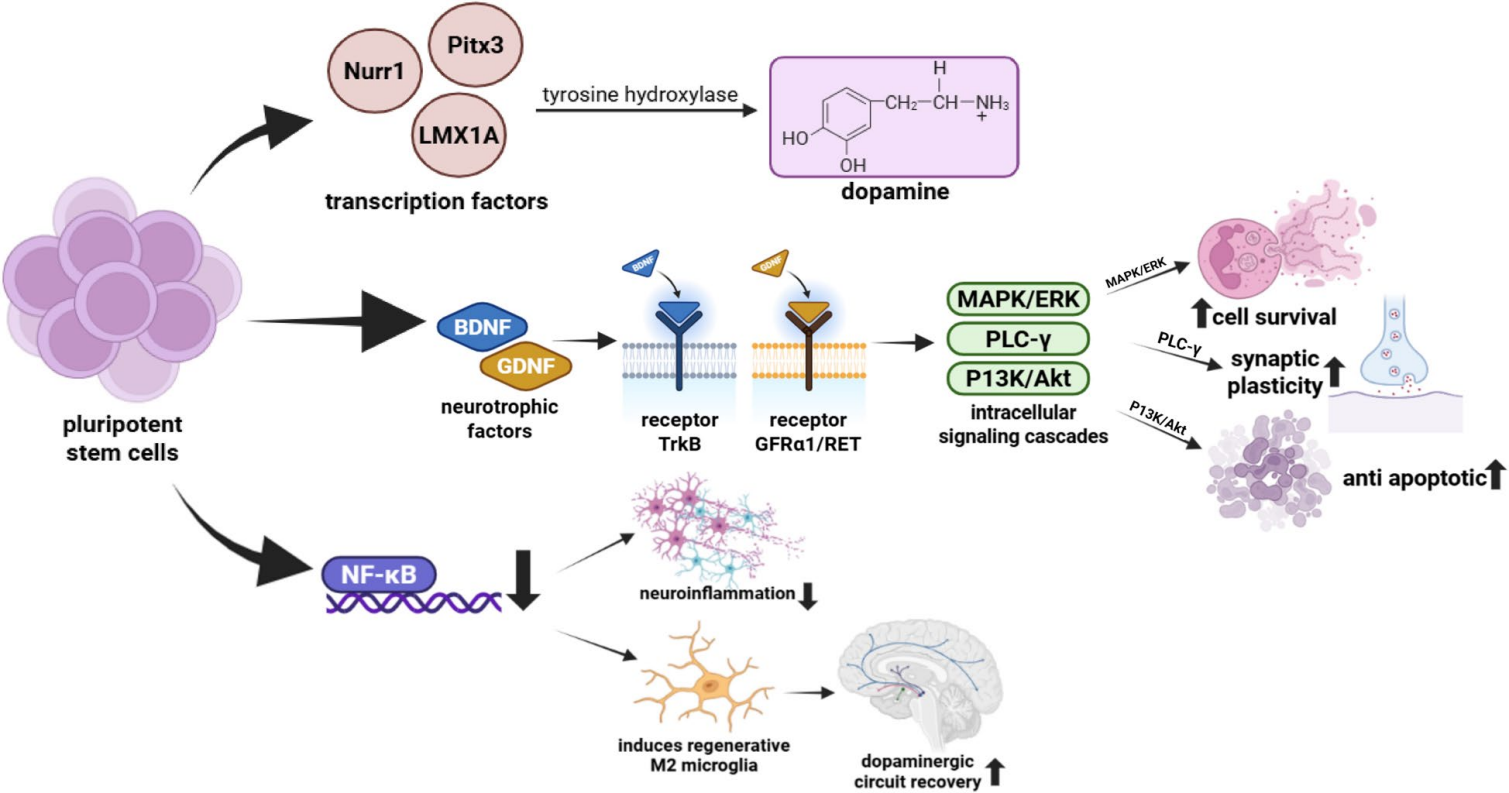
Mechanism of stem cell and BDNF

Brain-Derived Neurotrophic Factor exerts its effects in Parkinson’s disease at the cellular level via high-affinity binding to its receptor, TrkB. BDNF binding induces TrkB homodimerization and autophosphorylation at specific tyrosine residues, creating docking sites for adaptor proteins that trigger downstream signaling cascades [92]. Upon activation, TrkB initiates phosphorylation of specific tyrosine residues that recruit adaptor proteins, such as Src homology and Collagen (Shc) and Insulin Receptor Substrates 1 and 2 (IRS-1/2), setting off the PI3K/Akt, Ras/MAPK/ERK, and PLC-γ pathways. This is well-documented in neurotrophin signaling maps [93]. The PI3K/Akt cascade enhances neuronal survival by increasing anti-apoptotic proteins [e.g., B-Cell Leukemia/Lymphoma 2 (Bcl-2), B-Cell Lymphoma-extra large (Bcl-xL)] and inhibiting pro-apoptotic molecules [e.g.,Bcl-2-associated X protein (Bax), BCL2 Associated Agonist of Cell Death (Bad)], thereby protecting dopaminergic neurons from degeneration. BDNF–TrkB signaling has been shown to upregulate Bcl-2/Bcl-xL while reducing Bax activity [94]. Simultaneously, activation of the Ras/MAPK/ERK pathway promotes neuronal differentiation, synaptic plasticity, and gene transcription essential for neuron growth and maintenance. This MAPK-ERK branch is critical for plasticity and is a major downstream pathway of BDNF–TrkB [95]. The PLC-γ pathway is also engaged, elevating intracellular Ca^2^⁺ levels that in turn modulate neurotransmitter release, synaptic remodeling, and long-term potentiation. BDNF-induced PLC-γ activation is essential for Ca^2^⁺-dependent synaptic plasticity [96]. Collectively, these interconnected signaling pathways enhance the resilience, survival, and functional integration of dopaminergic neurons and may contribute to the restoration of damaged neural circuits in Parkinson’s disease. In PD models, reduced BDNF–TrkB signaling correlates with increased Bax/Bcl-2 ratio and neuron loss, while restoring this network improves neuronal survival and circuit maintenance [97]. Collectively, these mechanisms illustrate how combining stem cell therapy with BDNF enhances neuroregeneration and functional recovery in Parkinson’s disease, as summarized in Fig. 1.

Pluripotent stem cells can be directed to differentiate into dopaminergic neurons through the expression of transcription factors such as Nurr1, Pitx3, and LMX1A, which promote tyrosine hydroxylase activity and dopamine synthesis. Concurrently, BDNF and other neurotrophic factors (e.g., GDNF) bind to their respective receptors—TrkB and GFRα1/RET—initiating intracellular signaling cascades including MAPK/ERK, PLC-γ, and PI3K/Akt pathways. These cascades contribute to increased cell survival, enhanced synaptic plasticity, and anti-apoptotic effects, thereby supporting neuronal health and integration. Moreover, stem cell-derived therapies may attenuate neuroinflammation through the inhibition of NF-κB signaling, reducing the expression of proinflammatory cytokines. This downregulation favors the activation of M2 phenotype microglia, which are associated with a regenerative environment. Together, these effects facilitate the restoration of dopaminergic circuits in the brain, offering a promising neurorestorative strategy for Parkinson’s disease. The figure illustrates the convergence of cellular and molecular mechanisms that underpin the therapeutic synergy between stem cells and BDNF in neural repair.

The synergistic strategy combining stem cell transplantation with endogenous BDNF production addresses both loss of dopaminergic neurons and neurotrophic deficits in Parkinson’s disease. MSCs or iPSCs are engineered to stably express BDNF, thereby countering the reduced trophic support observed in PD models [65]. In this approach, MSCs or iPSCs are genetically modified—commonly via lentiviral vectors—to express BDNF continuously, enabling differentiation into dopaminergic progenitor-like cells and sustained BDNF release after transplantation. Studies show that human Umbilical Cord (hUC)-MSCs transduced to overexpress BDNF differentiate into DA-like neurons and secrete BDNF over time, improving motor outcomes in PD animal models [67]. Secreted BDNF binds to TrkB receptors on adjacent neurons, activating intracellular pathways such as PI3K/Akt and MAPK/ERK, which promote neuronal survival, enhance synaptic plasticity, and suppress apoptotic signaling. Experimental evidence links BDNF-modified MSC transplantation to upregulation of PI3K, phosphorylated Akt, and elevated TH/TrkB expression, correlating with neuroprotection [91]. Consequently, the combined effect of stem cell–derived dopamine progenitors and sustained BDNF signaling contributes to restoration of disrupted dopaminergic circuits and improvements in motor function in PD models. Behaviorally, rats receiving BDNF-modified MSCs exhibit reduced rotational behavior and increased dopamine levels in the striatum, reflecting functional recovery [98].

Despite the dual therapeutic advantages of stem cell transplantation combined with endogenous BDNF production—namely, cell replacement and sustained neurotrophic support—this strategy also faces significant drawbacks. These include the difficulty of tightly regulating BDNF levels to prevent excitotoxicity, potential immune reactions to genetically modified cells, and risks from viral vector–mediated gene insertion such as insertional mutagenesis and oncogenesis. One experimental strategy to minimize BDNF overexpression involves incorporating inducible gene expression systems, such as tetracycline-responsive promoters (Tet-On/Tet-Off), allowing temporal regulation of BDNF synthesis. Such systems enable controlled activation or suppression of transgene expression via administration of doxycycline, reducing excitotoxic risk [99]. Another approach employs advanced gene-editing tools like Clustered Regularly Interspaced Short Palindromic Repeats/CRISPR-Associated Protein 9 (CRISPR/Cas9) to precisely insert BDNF into “safe harbor” loci, minimizing off-target effects and lowering the risk of insertional mutagenesis. Although specific studies for BDNF in PD are emerging, the enhanced specificity of CRISPR/Cas9 is well-established as safer than random viral insertion vectors [100]. To further enhance safety, engineered stem cells can incorporate “suicide genes”—such as Herpes Simplex Virus Thymidine Kinase (HSV-tk)—which allow selective ablation upon administration of a prodrug in case of uncontrolled proliferation or adverse events. This switchable safety mechanism provides post-transplant regulation of modified cell populations [101, 102]. Together, these refinements—inducible promoters for tunable BDNF expression, precise CRISPR-based integration, and built-in safety switches—form a multi-layered control system that addresses the key risks of combined stem cell + BDNF therapy. Such a platform enhances therapeutic precision and safety, making the approach more viable for clinical translation.

## Conclusion

The combination of stem cell therapy and BDNF enhancement represents a promising and innovative direction in Parkinson’s disease treatment. By addressing both neuronal loss and trophic support, this synergistic approach offers the potential for not only improving symptoms but also modifying disease progression. With continued refinement and validation, this strategy could pave the way toward more effective, long-lasting therapies that bring hope to patients facing the challenges of Parkinson’s disease.

## Data Availability

All supporting data are included in this article. The cited information is derived from publicly available sources, appropriately referenced, and accessible through their respective original publications. For additional clarification or access to specific details, readers may contact the corresponding author.

## Abbreviations

Akt: Protein kinase B
APDA: American Parkinson Disease Association
BBB: Blood-brain barrier
BDNF: Brain-derived neurotrophic factor
Cas9: CRISPR-associated protein 9
CDNF: Cerebral dopamine neurotrophic factor
CRISPR: Clustered regularly interspaced short palindromic repeats
DA: Dopamine
DBS: Deep brain stimulation
ERK: Extracellular signal-regulated kinase
GDNF: Glial cell line-derived neurotrophic factor
GDP: Gross domestic product
GFRα1: GDNF family receptor alpha 1
hPSC: Human pluripotent stem cell
HSV-tk: Herpes simplex virus thymidine kinase
iPSC: Induced pluripotent stem cell
IRS: Insulin receptor substrate
LRRK2: Leucine-rich repeat kinase 2
MAPK: Mitogen-activated protein kinase
MAPK-ERK: Mitogen-activated protein kinase–extracellular signal-regulated kinase
MRI: Magnetic resonance imaging
MSC: Mesenchymal stem cell
mTOR: Mammalian target of rapamycin
NF-κB: Nuclear factor kappa-light-chain-enhancer of activated B cells
NGF: Nerve growth factor
NSC: Neural stem cell
PD: Parkinson’s disease
PI3K: Phosphoinositide 3-kinase
PLC: Phospholipase C
PSC: Pluripotent stem cell
REM: Rapid eye movement
RET: Rearranged during transfection
SHH: Sonic hedgehog
SNpc: Substantia nigra pars compacta
TH: Tyrosine hydroxylase
TrkB: Tropomyosin receptor kinase B
